# Trajectories of CVD medication after statutory retirement: contributions of pre-retirement sociodemographic, work and health-related factors: a register study in Finland

**DOI:** 10.1186/s12877-023-04272-8

**Published:** 2023-09-18

**Authors:** Jaakko Harkko, Olli Pietiläinen, Pekka Jousilahti, Ossi Rahkonen, Anne Kouvonen, Tea Lallukka

**Affiliations:** 1 Faculty of Social Sciences, University of Helsinki, Helsinki, Finland; 2 University of Helsinki, P.O. Box 20 (Tukholmankatu 8 B), N00014 Helsinki, Finland; 3 Department of Public Health, Faculty of Medicine, University of Helsinki, Helsinki, Finland; 4 Department of Public Health and Welfare, Finnish Institute for Health and Welfare, Helsinki, Finland; 5 Centre for Public Health, Queen’s University Belfast, Belfast, Northern Ireland

**Keywords:** Pharmaceuticals, Ageing, Registers, Retirement, Social determinants of health

## Abstract

**Background:**

Cardiovascular diseases (CVDs) are prevalent in older people, but few studies focus on developmental patterns in CVD medication directly after transition to statutory retirement.

We thus aimed to identify trajectories of CVD medication after retirement, and their sociodemographic, work and health-related determinants.

**Methods:**

We used complete register data of former employees of the City of Helsinki, Finland. All who reached their statutory retirement in 2000–2013, with five-year follow-up data (*n* = 6,505, 73% women), were included. Trajectories of CVD medication were identified with group-based trajectory modelling using data from Finnish Social Insurance Institution’s reimbursement register. Sociodemographic, work and health-related determinants of trajectory group membership were analysed using multinomial logistic regression.

**Results:**

Six trajectories of CVD medication were distinguished: "constant low" (35%), “late increase” (6%), “early increase” (5%), "constant high" (39%), "high and decreasing " (8%), and "low and decreasing” (7%). The majority (74%) of the retirees fell into the "constant low" and "constant high" categories. Lower occupational class and increased pre-retirement sickness absence were associated with the "constant high" trajectory. Further, those with lower educational attainment were more prone to be in the “early increase” trajectory.

**Conclusions:**

Individuals in lower socioeconomic positions or with a higher number of pre-retirement sickness absence may be considered at higher risk and might benefit from early interventions, e.g. lifestyle interventions and interventions targeting working conditions, or more frequent monitoring.

**Supplementary Information:**

The online version contains supplementary material available at 10.1186/s12877-023-04272-8.

## Background

Risk of cardiovascular disease (CVD) increases with age, and cardiovascular health is a significant issue in ageing societies despite huge improvements via better primary prevention, particularly reduction of smoking rates and the decrease of blood pressure and serum cholesterol levels [1]. CVDs are still the leading cause of death in Finland and globally both among women and men [2], but the onset of the disease has moved to older age, with differences by sex. In Finland, the probability of a 30-year-old men and women to die of CVD before their 70^th^ birthday has declined in the past 40 years from 30 to 7 percent among men and from 13 to 2 percent among women [3]. Health behaviours and socioeconomic position remain strong determinants of the risk of CVD, and contribute to the age of the CVD onset [4, 5].

Drugs used for prevention and treatment of CVDs are the most commonly used therapeutic classes of drugs in older adults [6]. While socioeconomic inequalities in cardiovascular health are well established particularly in working age adults [7, 8] less is known about the long-term effects of social determinants and the developmental patterns of cardiovascular health after transition to statutory retirement. A systematic review gathered results from 14 studies focusing on CVDs after retirement [9]. The included studies were mainly conducted in the United States or Europe, and the US ones typically did not find associations between retirement and CVD, whereas in the European studies except for one study from France, cardiovascular events, including CVD incidence or death, were consistently higher among retirees [9]. It is of note that these previous studies often focused on a dichotomous outcome, which fails to consider distinct developmental patterns, and their determinants. More specifically, most previous research has been variable-oriented, i.e. those using discrete outcome measures [10, 11]. These studies where the outcome is the first CVD medication purchase during the follow-up, using DDDs or a dichotomous variable, fail to detect different developmental patterns and risk groups and assume that people with CVD medication are a homogeneous group compared to those without medication. There is a paucity of research complementing previous studies using person-oriented methods, aiming to identify latent groups in CVD medication trajectories, and their pre-retirement determinants among retired people. It is important to examine how older individuals’ health develops in the long run, and what socioeconomic and other factors determine belonging to distinct trajectories. This may help identify new risk groups, as all people e.g., with a low socioeconomic position do not necessarily have similar risks or follow similar developmental patterns and benefit from same type of interventions.

Socioeconomic differences in CVDs are established during the entire life-course, from childhood pre-clinical changes to mid-adulthood risk factors and disease, as well as differences in older ages [12–15]. About half of the socioeconomic differences in the risk of CVD are attributable to known risk factors such as adverse health behaviours [16], but also access to care and cardiovascular deaths are socioeconomically shaped [17, 18].

The rationale for this study stems from the increasing prevalence of cardiovascular diseases (CVDs) among older populations, particularly those transitioning into retirement. Despite this trend, there is a scarcity of research examining the patterns of CVD medication use after retirement and how various sociodemographic, work and health-related determinants might impact these patterns. Understanding these patterns could provide valuable insights into the risk factors and how they evolve in the post-retirement phase.

The aim of this study was to identify developmental patterns in CVD medication during the first five years after transition to statutory retirement. Additionally, we aimed to examine sociodemographic, work and health-related determinants of the trajectory group membership. We first identified clusters of occurrence for CVD medication over a five-year follow-up period after retirement. Second, we investigated how pre-retirement sociodemographic, work and health-related determinants were associated with different CVD medication trajectories.

## Methods

### Population

This was a register-based cohort study. The study is a part of the Helsinki Health Study on health and well-being among employees of the City of Helsinki, Finland [19]. The City of Helsinki is the largest employer in Finland, with approximately 38,000 employees. The main occupational fields at the time of the participants’ careers were health and social care, education, social welfare services, public transport, and technical services. All employees of the City of Helsinki are covered by the same personnel administration, registration systems and policies including sickness absence and occupational health care.

The study population consisted of former employees of the City of Helsinki who transitioned to statutory retirement between 1^st^ January 2000 and 31^st^ December 2013 with at least five years of consecutive service prior to their retirement date (*n* = 6627, 73% women, representative of the population in question), and a five-year follow-up after their retirement (until 2018). Those who retired early due to disability were not included in this study. After excluding individuals with missing data on the study outcome variable (died during the first 1.5 years of the follow-up, *n* = 53), and those with missing baseline data on study variables (*n* = 69), the final analytic sample consisted of 6,505 participants. Death was a rare event (*n* = 110/1.7%), and those contributing the trajectories before that event were included. Data on retirement were obtained from the national registers of the Finnish Centre for Pensions, providing complete information on all retirement events.

### Outcome

The outcome was the purchases of prescribed, reimbursed CVD pharmacological medication. Purchases of these drugs, classified by the Anatomical Therapeutic Chemical Classification (ATC), were collected for the following groups: C01-C10 (excluding C05). These groups encompassed cardiac therapy (C01), antihypertensives (C02), diuretics (C03), peripheral vasodilators (C04), beta-blocking agents (C07), calcium-channel blockers (C08), agents affecting the renin-angiotensin system (C09), and lipid-modifying agents (C10). The follow-up time lasted five years, beginning from the first day after statutory retirement. Data were measured as dichotomous at six-month intervals producing ten (10) observations for each individual (positions t1…t10). The data were retrieved from the register of medical reimbursements from the Social Insurance Institution of Finland. This register includes all prescription medication purchases for all permanent residents in Finland.

To model developmental patterns in CVD medication during the five-year follow-up, we used six-month time windows. This allowed for a meaningful number of time points for the analyses. Medication can typically be bought for a three months use at a time, but summing the defined daily doses (DDDs) for six-month time windows was chosen for the modelling purposes, and as the medication is used continuously, the results are likely to be unaffected.

The Finnish healthcare system did not have any major changes during the recruitment period 2000–2013 nor the five-year follow-up until 2018. There were some changes in medication practices, mainly due to the development of new drugs. These drugs were included in the analyses. The therapeutic areas remained the same during the whole period and changes in the CVD care guidelines were also relatively small.

### Predictors

Different indicators were used to measure socioeconomic factors potentially associated with medication trajectories after retirement. Education, obtained from Statistics Finland’s registry, was classified into three levels: higher education (a Bachelor’s, Master’s or a doctoral degree), secondary education (high school, vocational school) and basic compulsory education. Occupational class was used as a proxy for physical and psychosocial working conditions. Data were derived from the personnel register of the City of Helsinki, including (i) managers (managerial and administrative work) and professionals (e.g., teachers and doctors), (ii) semi-professionals (e.g., nurses, foremen and technicians), (iii) routine non-manual employees (e.g., childcare workers and elderly care workers) and (iv) manual workers (e.g. transport and cleaning work).

Employment sector was coded as education (reference group), health and social care, or other. Working hours was the number of working hours per week in the last employment contract before the retirement and was coded as 0–34.9, 35–39.9 (reference group), and 40–44.8. We distinguished between full-time (reference group) and part-time jobs. Those with very few hours (0 to 10 h) were a small group (*n* = 506/7.8%), and mainly substitute teachers. All employment-related variables describe the situation of the last occupation during the employment and were retrieved from the City of Helsinki personnel register.

Data identifying baseline period sickness burden were measured with variables on sickness absence (SA) and retrieved from the City of Helsinki personnel register. We measured the yearly mean of total SA days (days per year) for all-cause SA during the five years before the retirement. The average number of sickness absence days per year was categorized into three groups: "low" (0–3.8 days), "intermediate" (3.9–9.4 days), and "high" (9.5–90.6 days). These cut-off points were determined with statistical software to create one baseline group and two groups of approximately equal size that reflect different levels of risk. For descriptive purposes, in Table 1, we present all CVD medication during the five years before retirement. The rationale for excluding this variable from statistical analyses was that independence between the dependent and independent variables is assumed in logistic regression analysis, and this assumption is not met in the case of these specific variables. The data were retrieved from the register of the Social Insurance Institution of Finland. Correspondingly with the outcome, the data were measured in six-month intervals for 4.5 years. The sum of the dichotomised observations was classified as 0, 1–6, and 7–9.

Other covariates used in the analysis were sex, age at the retirement (55–61, 62–63, 64–70) and the year of retirement divided into two periods (2000–2008 and 2009–2013). These were derived from the City of Helsinki personnel register. National ID numbers assigned to all permanent residents of Finland [20] were used to link the data from different registers. Table 1 further represents the characteristics of the study population by sex, as suggested by Sex and Gender Equity in Research (SAGER) guidelines. All data management procedures were performed with Stata 16.

### Statistical analysis

The development of CVD medication use before and after retirement was investigated using trajectory analysis [21]. We used group-based trajectory modelling (GBTM), which is an application of finite mixture modelling [22, 23]. The method identifies clusters of individuals, or trajectory groups, with an approximately similar developmental trajectory on a chosen outcome. The method assigns a subject to a trajectory group by assessing the probability of group membership. In this study, each time position (t1…t10) consisted of dichotomised (no/yes) data on having at least one purchase of CVD medication.

**Fig. 1:**
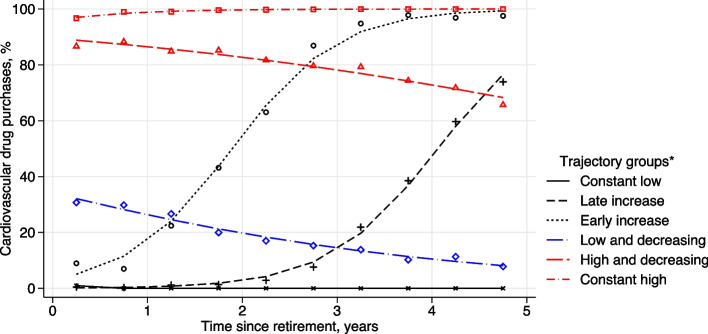
CVD medication trajectories after statutory retirement (k = 6)

The first step in model identification is the identification of a suitable number of trajectories. We applied the rules suggested by Nagin et al. [21]. The step involved estimating trajectory models with a varying number of groups. We held no assumptions of trajectories to follow any specific function of time. Therefore, we tested all possible polynomial combinations for each number of trajectory groups. Initially, we investigated whether convergence on the Bayesian Information Criterion (BIC) criteria could be achieved. We tested models with k = 2,…,7. BIC values did not converge, suggesting that the best model cannot be defined using BIC as criteria. We found the model consisting of six groups to best describe the data as in all models with k = 7, the group sizes fell below the suggested threshold of 5%. Thus, based on the distinctiveness and interpretability of the identified trajectory groups, a six group trajectory model provided the best description of the data and was selected. The selected model also held an average posterior probability of group membership above 70% and odds of correct classification above 5. The patterns were similar for men and women, and thus we present sex-specific figures as appendices (please see Online Appendices, Figs. 1 and 2).

Subsequently, multinomial logistic regression models were applied to investigate the sociodemographic, work and health-related differences in the trajectory group membership. The analyses compare each trajectory to the "constant low” category (reference category). In all these analyses, the estimates are given for sociodemographic, work and health-related variables, namely education, occupational class, employment sector, working hours per week, employment status, and sickness absence days. The small number of men in our sample with the initial finding that there was no association between sex and the outcome (data not shown) led us to analyse men and women in the same models while adjusting for sex in all the models. In addition to sex, all models were adjusted for age at the retirement and the year of retirement. The results are given as relative risk ratios (RRRs) with their 95% confidence intervals (CIs). All statistical analyses were performed with Stata 16 with the TRAJ command [24].

## Results

### Descriptive results

At the beginning of the follow-up, the study sample included 6505 former employees of the City of Helsinki, 1747 men and 4758 women, aged between 55 and 70. Table 1 provides the descriptive statistics for each variable in the analysis for men and women. In men, the unadjusted prevalence of CVD medication was highest in semi-professionals; whereas in women it was highest among manual workers. Those with a higher number of CVD drug purchases worked more often in the health and social care sector. There was a clear association between CVD drug purchases before and after retirement. Those with a high number of drug purchases during retirement had also more sickness absence days and CVD drugs purchases during the five years before retirement.

**Table 1:** Characteristics of the study population, *n* = 6505 [standard deviation, SD]

	Men		Women	
	N (%)	Number of periods with CVD drug purchases, % [SD]	N (%)	Number of periods with CVD drug purchases, % [SD]
All	1747 (100.0)	5.24 [4.54]	4758 (100.0)	5.03 [4.53]
Age				
55–61	433 (24.8)	5.19 [4.51]	1425 (29.9)	4.69 [4.52]
62–63	916 (52.4)	5.29 [4.55]	2165 (45.5)	5.14 [4.54]
64–70	398 (22.8)	5.20 [4.54]	1168 (24.5)	5.23 [4.51]
Year of retirement				
2000–2008	1002 (57.4)	5.04 [4.55]	2529 (53.2)	4.83 [4.55]
2009–2013	745 (42.6)	5.51 [4.52]	2229 (46.8)	5.26 [4.50]
Education				
Higher education	797 (45.6)	5.02 [4.58]	1944 (40.9)	4.71 [4.53]
Secondary education	454 (26.0)	5.65 [4.47]	1496 (31.4)	5.22 [4.50]
Basic education	496 (28.4)	5.24 [4.52]	1318 (27.7)	5.29 [4.54]
Occupational class				
Managers or professionals	652 (37.3)	5.07 [4.58]	1216 (25.6)	4.43 [4.48]
Semi-professionals	350 (20.0)	5.42 [4.58]	881 (18.5)	5.16 [4.55]
Routine non-manual workers	130 (7.4)	5.24 [4.61]	1946 (40.9)	5.17 [4.53]
Manual workers	615 (35.2)	5.33 [4.46]	715 (15.0)	5.52 [4.50]
Employment sector				
Education	204 (11.7)	4.99 [4.56]	620 (13.0)	4.45 [4.50]
Social and health	184 (10.5)	5.52 [4.55]	2531 (53.2)	5.24 [4.52]
Other	1359 (77.8)	5.25 [4.54]	1607 (33.8)	4.93 [4.54]
Working hours				
0–34.9	577 (33.0)	5.23 [4.53]	1598 (33.6)	5.11 [4.53]
35–39.9	1043 (59.7)	5.36 [4.53]	2898 (60.9)	5.04 [4.53]
40.0–44.8	127 (7.3)	4.31 [4.57]	262 (5.5)	4.44 [4.51]
Employment status				
Full-time	1252 (71.7)	5.26 [4.55]	3452 (72.6)	4.87 [4.53]
Part-time	495 (28.3)	5.20 [4.51]	1306 (27.4)	5.44 [4.51]
Prior CVD drug purchases in 6 months periods				
0	800 (45.8)	1.37 [2.69]	2197 (46.2)	1.24 [2.49]
1.0–6.0 (0.5–3 years)	372 (21.3)	7.24 [3.70]	1086 (22.8)	6.79 [3.94]
7.0–9.0 (3.5 years-4.5 years)	575 (32.9)	9.35 [1.82]	1475 (31.0)	9.38 [1.69]
Sickness absence days, yearly average				
Low	1082 (61.9)	4.79 [4.57]	2194 (46.1)	4.36 [4.50]
Intermediate	353 (20.2)	5.76 [4.46]	1255 (26.4)	5.27 [4.54]
High	312 (17.9)	6.21 [4.31]	1309 (27.5)	5.92 [4.40]

### CVD medication trajectories

A trajectory model consisting of six distinct trajectories showed the best fit using the BIC criterion, model stability and interpretability in trajectory analysis. Figure 1 shows the trajectories where three of them represent trajectories with low levels of drug purchases at the beginning of the follow-up (in black colour). They represented about 45% of the whole study population and were labelled as "constant low" (35%), indicating a consistent and sustained low level throughout the observed period, "late increase" (6%) suggesting a relatively low level initially, followed by a notable increase occurring later in the observed period, and "early increase" (5%) signifying a relatively low level initially, followed by a significant increase early on in the observed period. Two trajectories representing 47% of the population initiated from high starting values (in red colour). They were labelled as "constant high" (39%), indicating a consistently high level maintained throughout the observed period and "high and decreasing" (8%), suggesting a high level initially, followed by a gradual decrease over the observed period. One trajectory representing 7% of the study population had low starting values with decreasing trajectory and was labelled "low and decreasing”.

### Contributions of pre-retirement health and work-related factors

The results from the multinomial regression models are shown in Table 2. The most consistent association between pre-retirement work and health-related factors with post-retirement medication trajectories was found for the "constant high" trajectory. Each studied variable was statistically significantly associated with this trajectory. The likelihood of belonging to this trajectory was increased for those having lower educational credentials, being in lower occupational classes when compared with the employees in managerial or professional positions, working in the health and social care sector, and working part-time prior to retirement. Working long hours (> 40 h / week) was not associated with belonging to this trajectory. More specifically, secondary and basic education were associated with higher odds of belonging to the trajectories of “early increase”, and “constant high”, and lower secondary education was also associated with higher odds of belonging to the trajectory group “low and decreasing” (Table 2).

**Table 2:** Pre-retirement sociodemographic, work and health-related factors and post-retirement CVD medication trajectories, relative risk ratios (RRRs) and their 95% confidence intervals (95% CIs) in multinomial logistic regression. Analyses vs. the Constant low trajectory (*n* = 2263, 34.8%)

	Late increase^a^	Early increase^a^	Low and decreasing^a^	High and decreasing^a^	Constant high^a^
n (%)	400 (6.2%)	331 (5.1%)	424 (6.5%)	531 (8.2%)	2556 (39.3%)
	RRR (95% CI)	RRR (95% CI)	RRR (95% CI)	RRR (95% CI)	RRR (95% CI)
Education					
Upper secondary or higher	1	1	1	1	1
Lower secondary	1.25 (.97–1.61)	1.39 (1.05–1.85)	1.55 (1.20–2.00)	1.11 (.87–1.41)	1.36 (1.19–1.56)
Basic education	1.20 (.93–1.55)	1.41 (1.06–1.88)	1.04 (.77–1.38)	1.07 (.84–1.37)	1.26 (1.09–1.44)
Occupational class					
Managers or professionals	1	1	1	1	1
Semi-professionals	1.07 (.78–1.45)	.83 (.57–1.20)	1.20 (.86–1.67)	1.14 (.85–1.53)	1.39 (1.17–1.64)
Routine non-manual workers	1.01 (.82–1.41)	1.23 (.90–1.67)	1.28 (.95–1.72)	1.02 (.78–1.33)	1.37 (1.18–1.60)
Manual workers	1.18 (.87–1.60)	1.33 (.95–1.86)	1.29 (.93–1.79)	1.29 (.97–1.71)	1.46 (1.24–1.72)
Employment sector					
Education	1	1	1	1	1
Health and social care	1.15 (.82–1.60)	1.21 (.83–1.75)	1.14 (.80–1.63)	1.20 (.87–1.65)	1.43 (1.19–1.72)
Other	1.09 (.78–1.52)	1.03 (.71–1.50)	1.25 (.87–1.79)	1.10 (.80–1.51)	1.24 (1.03–1.49)
Working hours per week					
0–34.9	.91 (.73–1.14)	1.02 (.79–1.30)	.91 (.72–1.16)	1.07 (.87–1.32)	.98 (.86–1.10)
35–39.9	1	1	1	1	1
> 40	.62 (.31–1.26)	.95 (.48–1.87)	1.34 (.76–2.34)	.67 (.34–1.31)	.70 (.50-.99)
Employment status					
Full-time	1	1	1	1	1
Part-time	1.03 (.80–1.32)	1.08 (.82–1.43)	1.09 (.84–1.40)	1.24 (.99–1.55)	1.18 (1.04–1.35)
Sickness absence days, yearly average					
Low	1	1	1	1	1
Intermediate	1.35 (1.04–1.74)	1.51 (1.14–1.99)	1.34 (1.01–1.77)	1.19 (.93–1.54)	1.66 (1.45–1.91)
High	1.55 (1.19–2.02)	1.50 (1.10–2.04)	2.20 (1.68–2.87)	2.00 (1.58–2.55)	2.45 (2.12–2.83)

^a^All models were adjusted for sex, age at the retirement and the year of retirement

Also the measure on pre-retirement sickness absence was associated with the trajectories of CVD medication purchases after retirement.

## Discussion

### Main findings

In this study we sought to examine the distinct developmental patterns in CVD medication after statutory retirement; and the pre-retirement sociodemographic, work and health-related determinants of these trajectories. We identified six distinct trajectories of CVD medication among retired, former public sector employees. The two largest trajectories, constant low (35%) and constant high (39%), represented three quarters of the population of statutory retirees of the City of Helsinki, Finland. Furthermore, we found that the assignment to these groups was attributable to both socioeconomic and health determinants before retirement. Those with a lower educational level, lower occupational class, those working in the health and social care sector, and those with part-time contracts had higher odds of belonging to the constant high trajectory group. Working over 40 h per week lowered the odds. We also identified four patterns of change in CVD medication after retirement: “late increase”, “early increase”, “low and decreasing”, and “high and decreasing”. Those with lower educational credentials were more likely to belong to the early increase trajectory group. Pre-retirement sickness absence spells were associated with the constant high trajectory and the all four trajectories indicating changes in medication after retirement.

### Interpretation

As a special novelty value, we could follow-up people from the date of their actual confirmed retirement. Thus, we could produce new information about the development of cardiovascular health – in our case CVD medication – during the first five years after retirement. This is of importance, as many earlier studies either focus on risk factors of CVD or medication among working-age people, or focus on elderly people, who may have already spent a number of years as retirees, and who could be more fragile and have many other health conditions. The present results increase our understanding on healthy ageing, and the third age, and how cardiovascular health develops directly after retirement. Moreover, we could show how pre- retirement socioeconomic and work-related factors are associated with the development of cardiovascular health.

It is of note that while our follow-up started after retirement, when participants were over 60 years of age, the development of CVD takes typically decades. As our cohort is female-dominated (80%), sex differences must also be considered. CVD is often diagnosed later among women than men, and there is a clear male dominance before the age of 80, after which this reverses, and the excess CVD deaths are seen in women [2]. As our cohort is following up people directly after their retirement, the determinants are from years immediately preceding the retirement where there are the most striking differences in disability adjusted life years for men as compared to women (ages 30 and 60 years) [2]. In older age groups, we may further expect to see larger absolute differences regarding key risk factors such as smoking, while relative risks could be similar [25]. This is because with ageing, health deteriorates with or without the presence of key risk factors. The identified trajectories in the current study support this, because the associations with established risk factors (socioeconomic factors) were the weakest for those who belonged to the trajectory of “late increase” (indicating late onset disease), slightly stronger for those who belonged to the trajectory of “early increase” (indicating diagnosis shortly after retirement), and the strongest for those who belonged to the trajectory showing high medication early (diagnosed during working age).

As the development of CVD is a long process, the prevention has to start early, to detect early risk factors and their causes. In particular, it is important to consider socioeconomic inequalities and the role of social determinants of CVDs. This means that the focus cannot only be on individuals but on society and societal level and structures. If we are to successfully prevent CVDs, it is essential that the environments and societies are health promoting. This is also a more effective way to prevent illness, if we can prevent diseases in populations, as compared to typically short-term individual level interventions which have often failed to produce long-term favorable changes in key pertinent risk factors of CVDs, including smoking, physical activity, unhealthy nutrition and obesity [1, 26]. Thus, it is often difficult to target the interventions on those who would mostly benefit from them, but in the worst case, individual interventions even widen the inequalities, if those individuals with the highest education and those who are already better off benefit from them more [27]. Identifying distinct developments could also help better target policy interventions, as it is likely that all groups do not need similar interventions, and some groups may not even need them at all. Furthermore, it is important to pay special attention to those groups that are more disadvantaged and often the hardest to reach [28].

As we focus on changes in health as indicated by CVD medication directly after retirement, it is important to consider potential explanations for the identified distinct patterns in the CVD medication development. Firstly, an important change is that there is no longer occupational health care available. In Finland, practically all employees have access to occupational health care free of charge for them. After retirement, such fast access to health care is not similarly available, which could explain some of the patterns. Employees are allowed to request a health check-up before they retire, but this is not mandatory. In such check-ups, a topic can be about continuation of the treatment, where necessary, and this could be reflected in the development of health during our follow-up.

After retirement, another important change is change in income, which alongside not having access to occupational health care, could affect the opportunities to maintain good health. However, it could be seen as somewhat surprising that low education was associated with higher odds of belonging to the trajectory of “early increase”. Lower educated people may have retired as soon as they could using statutory retirement scheme, whereas those in higher socioeconomic positions may have remained working as long as they were allowed. This may support the theory of accumulation of disadvantages, however, this remains speculative.

Instead, it is unlikely that deaths could explain the “early decrease”. This group existed in the initial analyses, when all those who died during the follow-up were excluded (data not shown). It also needs to be noted that the group, as all trajectory groups, are approximations and they may or may not describe the true development.

Furthermore, one could assume that”decrease” could be due to healthy changes during follow-up, such as removal of physically and mentally poor working conditions or work-related stress, having more time to exercise, changes in diet, etc., but we lack data to confirm these suggestions. One could also assume that the CVD medication might decrease even though it would be still needed with decreasing income or decreasing ability to take care of one’s health. Additionally, medication use may decrease with approching death, if the medication is deemed as non-essential, but as our group is still relatively young and the follow-up concerns the first five years after retirement, this explanation is unlikely.

### Methodological considerations

Some limitations and strengths have to be considered that affect how to interpret the findings and their generalisability. First, we had no information on behavioural risk factors such as smoking, alcohol use, physical activity, food consumption, sleep or obesity available that are likely to contribute to the developmental trajectories of CVD medication, due to their established links to CVDs [1, 16]. Nonetheless, all these risk factors are strongly patterned by socioeconomic position [16], which we considered. Indeed, the included indicators of socioeconomic position are likely to be behind of these behavioural risk factors, acting as causes of causes [29]. Similarly, we could not consider working conditions such as job strain, which is strongly linked to CVDs [30], but also linked to socioeconomic position [31]. Another limitation is that the results cannot be generalised to the entire population, as our highly female-dominated cohort was comprised people with a long stable employment history, as compared to e.g., unemployed people or people with more precarious jobs. Additionally, our study design has potential sources of selection bias. Individuals with poorer health may have adapted to their condition by transitioning to part-time work or otherwise reducing their working hours rather than these factors acting as determinants of subsequent health. Not having data on comorbidities and chronic conditions can also be considered a limitation. Furthermore, those with the poorest health have most likely exited paid employment due to disability, a route not included in this study. Those with a lower socioeconomic position are likely overrepresented in that route of exit. Moreover, death during follow-up was a relatively rare event, probably again due to the fact that the cohort was female-dominated and retired rather young, and as we excluded retirement due to disability (illness). The average statutory retirement age was in this population 62.4 years. During the 5-year follow-up, deaths were the most common event among those who retired at age 63, and in relative terms, among those who retired even younger (further data not shown). This supports the assumption that health is a clear determinant of not only the route but also of age and timing of the retirement.

Future studies with large survey data could extend from our findings and examine further the key predictors of distinct CVD medication trajectories, including effect of comorbidities, chronic conditions, working conditions and lifestyle factors. Future studies could also explore whether the transition to retirement affects CVD trajectories.

Our study had several strengths. First, the data were large and comprehensive, as we could include all who had been employed by the City of Helsinki during the study period, and we could also include objective information about their medication use before and after the statutory retirement date. The data on medication were based on prescribed, reimbursed medication, which is more reliable than self-reports. Second, we could study the development in CVD medication over several years in short time windows, which helped identify distinct developmental patterns and the determinants of different development. The group-based trajectory models allow for examining potential factors influencing medication patterns. This approach helps uncover patterns that may be missed in discrete analyses and, by doing so, offers insights into the dynamic nature of medication use. Third, the personnel register further comprised information about factors such as prior sickness absence and working hours before retirement. Fourth, the City of Helsinki is the largest employer in Finland, and represents hundreds of different occupational titles from manual work to administrative and executive level jobs.

## Conclusion

We identified six distinct trajectories of CVD medication after statutory retirement among former public sector employees. Socioeconomic factors and health before retirement were associated with the distinct developmental patterns in CVD medication, with the strongest associations found for those belonging to the constant high trajectory, i.e., the trajectory of earlier onset of CVD. These findings underscore the need for tailored early prevention strategies targeted at working-age individuals who belong to high-risk groups. The results enrich our understanding of post-retirement CVD medication patterns and offer valuable insights for shaping proactive and targeted healthcare interventions, potentially altering the course of CVD progression in at-risk working populations. Individuals in lower socioeconomic positions or with a higher number of pre-retirement sickness absence may be considered at higher risk and might benefit from early interventions or more frequent monitoring during their working life and after retirement.

### Supplementary Information

**Additional file 1:** **Appendix Figure 1.** CVD medication trajectories after statutory retirement (k= 6) for men.

**Additional file 2:** **Appendix Figure 2.** CVD medication trajectories after statutory retirement (k= 6) for women.

## Data Availability

The data are available from the register data holders only, pending their approval and a research plan. Data cannot be openly shared doe to data protection laws.

## Abbreviations

ATC: Anatomical Therapeutic Chemical Classification
BIC: Bayesian information criterion
CVD: Cardiovascular diseases
CI: Confidence intervals
DDDs: Defined daily doses
GBTM: Group-based trajectory modelling
RRR: Relative risk ratios
SAGER: Sex and gender equity in research
SA: Sickness absence
SD: Standard deviation
